# Comparative analysis of autonomic nervous system function in patients with postural orthostatic tachycardia syndrome versus a head-up tilt testing-negative cohort

**DOI:** 10.1016/j.ibneur.2025.04.002

**Published:** 2025-04-04

**Authors:** Jing-Xiu Li, Xin Qiu, Min Gao

**Affiliations:** a Department of Electrocardiogram, The First Affiliated Hospital of USTC, Division of Life Sciences and Medicine, University of Science and Technology of China, Hefei, Anhui Province, China

**Keywords:** Postural orthostatic tachycardia syndrome, Head-up tilt test, Autonomic dysfunction, Heart rate variability

## Abstract

**Purpose:**

Postural orthostatic tachycardia syndrome (POTS) is a heterogeneous collection of disorders that correspond to autonomic dysfunction. Until recently, it was mostly unresolved as a clinical issue. Our study investigated autonomic function in patients with POTS.

**Methods:**

The research utilizes both pre-test 24-hour Holter monitoring to evaluate heart rate variability (HRV) and instantaneous HRV during the head-up tilt test (HUTT), aiming to discern differences in autonomic nervous system (ANS) function between patients positively diagnosed with POTS and those exhibiting negative HUTT results.

**Results:**

The 24-hour Holter results derived from time-domain methods demonstrated that SDNN, SDANN, SDNN index, rMSSD, pNN50, and TINN were significantly elevated in the POTS positive group compared to the HUTT-negative group. Meanwhile, frequency-domain methods revealed that low-frequency and high-frequency were significantly elevated in the POTS-positive group relative to the HUTT-negative group. However, the analysis of instantaneous time-domain and frequency-domain parameters during HUTT, including SDNN, RMSSD, SDSD, PNN50, VLF, LF, and HF, revealed no statistically significant differences.

**Conclusion:**

The findings suggest that pre-HUTT HRV, as assessed by 24-hour Holter monitoring, may provide greater clinical relevance in distinguishing between these groups. The study reveals that patients with POTS demonstrate abnormalities in autonomic regulation, characterized by a decrease in sympathetic activity alongside an increase in parasympathetic activity. The dysregulation of autonomic balance likely contributes to the elevated incidence of syncope and presyncope events observed in patients with POTS.

## 1 Introduction

Postural Orthostatic Tachycardia Syndrome (POTS) represents the most prevalent form of orthostatic intolerance. Its diagnosis is established based on a sustained heart rate (HR) increase exceeding 30 beats per minute, or a heart rate reaching 120 beats per minute, in the absence of accompanying arterial hypotension (Stewart et al., 2018). Approximately 3,000,000 Americans are afflicted by this condition, with a female-to-male ratio of 4–5:1 (Boris, 2018). It is frequently under-recognized or misdiagnosed due to nonspecific and overlapping symptoms; consequently, the prevalence may be even higher. It primarily occurs in children and young adults aged 14–45 years (Swai et al., 2019).

The autonomic nervous system (ANS) plays a crucial role in regulating hemodynamics and preventing conditions like POTS (Lambert and Lambert, 2014). The term "autonomic nervous system" was first introduced by John Newport Langley in 1898, who also described the roles of its sympathetic and parasympathetic components (Maehle, 2004). Heart rate variability (HRV) is a specific, noninvasive electrocardiographic measure of ANS function, reflecting both sympathetic and parasympathetic activity. HRV can be assessed using two primary approaches: time-domain measures (TDM) and frequency-domain measures (FDM). The time-domain heart rate variability (HRV) parameters include several key metrics: the standard deviation of all normal-to-normal (NN) intervals (SDNN), the standard deviation of the mean 5-minute NN intervals (SDANN), the mean of the standard deviations of all NN intervals across 5-minute segments over a 24-hour period (SDANN index), the root mean square of successive differences between adjacent normal cycles (rMSSD), and the percentage of NN intervals differing by more than 50 ms in relation to the total number of NN intervals (pNN50). In addition, frequency-domain HRV parameters encompass very low frequency (VLF), low frequency (LF), high frequency (HF), and the LF/HF ratio (Anon, 1996; Li et al., 2023).

POTS was first documented in the medical literature by Dr. Schondorff and Dr. Low (Schondorf and Low, 1993). Despite its recognition, research on POTS remains limited. The pathophysiology of POTS is multifactorial including impaired sympathetically mediated vasoconstriction, excessive sympathetic nervous system activity, dysregulation of blood volume, and deconditioning. It is important to emphasize that patients with indirect evidence of peripheral sympathetic denervation in the lower limbs are classified as having neuropathic POTS. This subset of patients demonstrates impaired sympathetic vasoconstriction, as evidenced by abnormal blood pressure profiles during HUTT, and experience venous pooling in the lower extremities. This study aims to analyze HRV parameters between patients diagnosed with POTS and those with syncope or presyncope, in cases where HUTT yields negative results.

## 2 Methods

### 2.1 Patients and ethics

A retrospective analysis of historical data from January 1, 2017, to December 12, 2024, identified 26 individuals who experienced syncope or presyncope at the First Affiliated Hospital of the University of Science and Technology of China (USTC). Among these, 13 were diagnosed with POTS, while the remaining 13 had negative results on the HUTT. Thyroid function tests were conducted to exclude the presence of hyperthyroidism or hypothyroidism. Ethical approval for this study was granted by the ethics committee of the First Affiliated Hospital of USTC, in accordance with established ethical guidelines, including the Declaration of Helsinki. Written informed consent was obtained from all participants [2024-RE-468].

### 2.2 Enrollment criteria and exclusion criteria

The primary inclusion criteria were as follows: (1) recurrent episodes of unexplained syncope, with structural heart disease and cardiogenic syncope explicitly excluded, and (2) evaluation for the presence of postural orthostatic tachycardia syndrome.

The diagnostic criteria for POTS are as follows: (1) an increase in heart rate exceeding 30 beats per minute in adult (or more than 40 beats per minute in children aged 12–19 years) or a heart rate exceeding 120 beats per minute following a positional change from HUTT within 10 minutes of movement; and (2) the absence of orthostatic hypotension, defined as a decrease in systolic blood pressure of at least 20 mmHg and/or diastolic blood pressure of at least 10 mmHg.

The exclusion criteria were as follows: (1) evaluation of the therapeutic effects in patients with syncope; (2) syncope-related falls resulting in severe adverse events or unstable medical conditions; (3) the presence of significant vascular or cardiac conditions, including severe intracranial or extracranial vascular stenosis, severe coronary artery stenosis, severe aortic or mitral valve stenosis, or severe hypertrophic obstructive cardiomyopathy; (4) severe anemia; (5) severe arrhythmias; (6) moderate to severe hypertension; (7) pregnancy; (8) hyperthyroidism; and (9) incomplete or insufficient patient data.

### 2.3 Measurement of heart rate variability

Heart rate variability (HRV) metrics are generally categorized into time-domain and frequency-domain measures (BaiHui, CT-82). Time-domain metrics are derived over a 24-hour period directly from the patient’s heart rate or from intervals between successive RR waves. Frequency-domain measures are obtained over 24 hours through spectral analysis of ECG recordings. In accordance with the recommendations of the Task Force and the frequency of reporting, the selected time-domain HRV parameters included SDNN, SDANN, the SDNN index, RMSSD, and pNN50. Additionally, one frequency-domain measure was examined, with the very low-frequency (VLF), low-frequency (LF), high-frequency (HF), and LF/HF ratio statistically recorded and analyzed. The HRV indicators mentioned were assessed through the integrated measurement features of 24-hour Holter monitoring (BaiHui, CT-82). Enrolled patients were instructed to refrain from engaging in intense physical exercise, consuming alcohol, and smoking. They were advised to cease physical activities by 10 p.m. and maintain a sleep schedule from 10 p.m. to 6 a.m. All examinations were conducted within the controlled environment of our hospital to minimize the impact of potential confounding factors, including occupational stress and dietary variations.

Furthermore, we conducted a comprehensive analysis of real-time heart rate variability (HRV) throughout the head-up tilt test (HUTT) process, utilizing key metrics including SDNN, RMSSD, SDSD (standard deviation of successive differences), PNN50, very low frequency (VLF), low frequency (LF), high frequency (HF), and the LF/HF ratio. The aforementioned indicators were assessed using the integrated measurement capabilities of the equipment system (STandard SHUT-100).

### 2.4 Measurement of head-up tilt test

All tests were conducted in a quiet, dimly lit room to ensure a controlled environment. Patients were instructed to abstain from coffee consumption and smoking prior to testing. After being positioned supine on the testing table, a blood pressure cuff and electrocardiogram (ECG) electrodes were carefully placed at appropriate anatomical sites. Blood pressure was measured using a cuff applied to the patient’s right arm. The HUTT protocol (STandard SHUT-100) included a 10-minute supine phase followed by a 30-minute tilted phase, with an angle of 60 degrees for children and 70 degrees for adults. The table was tilted gradually from 0 to 60/70 degrees over a 15-second period. Should syncope not manifest, for patients aged over 14 years, a pharmacological provocation phase is commenced. This involves the administration of sublingual nitroglycerin (300 µg), a vasodilator, to facilitate the identification of neurally mediated syncope. Subsequent recovery is monitored while the patient is in the supine position. Blood pressure and heart rate measurements were recorded throughout the procedure using standardized head-up tilt test equipment.

The diagnostic criteria for POTS stipulate an initial 10-minute rest in the supine position, followed by a heart rate increase exceeding 30 beats per minute in adults (or more than 40 beats per minute in children aged 12–19 years), or a heart rate exceeding 120 beats per minute upon tilting the patient to an angle of 60 degrees for children and 70 degrees for adults within 10 minutes of positional change during HUTT, all in the absence of orthostatic hypotension, which is defined as a decrease in systolic blood pressure of at least 20 mmHg and/or a decrease in diastolic blood pressure of at least 10 mmHg.

### 2.5 Statistical analysis

Data analysis was conducted using IBM SPSS Statistics software (Version 27.0, IBM Corporation, Chicago, IL, USA). The Shapiro-Wilk test was employed to assess the normality of the data distribution. Continuous variables with a normal distribution were expressed as mean ± standard deviation, whereas those not conforming to a normal distribution were reported as median and interquartile range. Categorical variables were analyzed using Pearson’s chi-squared test.

## 3 Results

Between January 1, 2017, and December 12, 2024, a total of 26 patients presenting with syncope or presyncope at our hospital were enrolled and categorized into two groups: the POTS-positive group (Group 1) and the HUTT-negative group (Group 2). Of the 26 participants included in this observational study, 13 were male, yielding a male-to-female ratio of 1:1. The mean age was 16.08 ± 2.19 years in Group 1 and 15.77 ± 2.65 years in Group 2. Based on the data obtained (Table 1), statistical analysis revealed no significant differences between the two groups in terms of age, systolic blood pressure, diastolic blood pressure, serum potassium levels, glucose levels, hemoglobin concentrations, or ejection fraction. All patients underwent 24-hour ambulatory electrocardiography pre-HUTT to evaluate heart rate variability, as well as minimum, mean, and maximum heart rate.

**Table 1:** Baseline Characteristics of the Included Patients Enrolled in this study (n = 26).

	Group1	Group 2	T-test/χ^2^ test	P value
Male (n, %)	6 (46.15)	7 (53.85)	0.15	0.70
Age (year)	16.08 ± 2.19	15.77 ± 2.65	0.09	0.93
SBP (mmHg)	113.77 ± 3.35	116 ± 4.41	0.4	0.69
DBP (mmHg)	76.15 ± 3.17	69.17 ± 3.45	1.49	0.15
Potassium	4.08 ± 0.08	4.1 ± 0.07	0.24	0.81
Glucose	5.07 ± 0.43	4.75 ± 0.14	0.7	0.49
Hemoglobin	129.15 ± 3.87	137.25 ± 3.31	1.59	0.13
EF (%)	70 ± 1.16	68.09 ± 1.68	0.93	0.36

Group 1: patient with postural orthostatic tachycardia syndrome. Group 2: Patients experiencing syncope or presyncope exhibited negative results on the head-up tilt test.

Systolic blood pressure (SBP), diastolic blood pressure (DBP), and ejection fraction (EF) are indicated.

The 24-hour Holter results based on time-domain methods indicated that SDNN (150.23 ± 11.57 vs. 114.77 ± 7.02, P < 0.05), SDANN (129.69 ± 10.28 vs 102.23 ± 7.31, P < 0.05), SDNN index (69.85 ± 5.35 vs. 50.46 ± 3.06, P < 0.05), rMSSD (50.62 ± 5.82 vs. 27.85 ± 2.65, P < 0.05), pNN50 (20.08 ± 3.26 vs. 8.27 ± 1.77, P < 0.05), TINN (41.31 ± 4.08 vs. 30.91 ± 2.24, P < 0.05) were significantly higher in patients with POTS positive group compared to the HUTT-negative group (Table 2).

**Table 2:** Time-Domain and Frequency-Domain Parameters Over 24 Hours in POTS Patients and Those with Syncope or Presyncope with Negative Head-Up Tilt Test Results.

	Group1	Group 2	T-test/χ^2^ test	P value
Max HR (bpm)	154.08 ± 3.62	148.31 ± 3.24	1.19	0.25
Min HR (bpm)	52.23 ± 2.18	57.62 ± 1.59	2	0.06
Mean HR (bpm)	83.38 ± 2.55	89.08 ± 1.76	1.84	0.08
SDNN (ms)	150.23 ± 11.57	114.77 ± 7.02	2.62	0.02
SDANN (ms)	129.69 ± 10.28	102.23 ± 7.31	2.18	0.04
SDNN index (ms)	69.85 ± 5.35	50.46 ± 3.06	3.14	0.01
rMSSD (ms)	50.62 ± 5.82	27.85 ± 2.65	3.56	< 0.05
pNN50 (%)	20.08 ± 3.26	8.27 ± 1.77	3.19	< 0.05
TINN (ms)	41.31 ± 4.08	30.91 ± 2.24	2.23	0.04
LF/HF	1.14 ± 0.19	1.73 ± 0.44	1.25	0.23
LF (ms^2^)	1163.99 ± 152.01	561.07 ± 83.33	3.48	< 0.05
HF (ms^2^)	1347.06 ± 242.42	468.19 ± 91.25	3.39	< 0.05

Group 1: patient with postural orthostatic tachycardia syndrome. Group 2: Patients experiencing syncope or presyncope exhibited negative results on the head-up tilt test.

The standard deviation of all normal to normal NN intervals (SDNN), standard deviation of all mean 5-minute NN intervals (SDANN), mean of the standard deviation of all NN intervals for all 5-min segments over 24 hours (SDNN index), root mean square of successive differences between adjacent normal cycles (RMSSD), and percentage of NN 50 in the total number of NN intervals (PNN50) are indicated. TINN: triangular interpolation of the NN interval histogram. LF: low frequency, HF: high frequency, LF/HF: ratio of low frequency and high frequency.

The 24-hour Holter recordings with frequency-domain methods indicated that LF (1163.99 ± 152.01 vs. 561.07 ± 83.33, P < 0.05), and HF (1347.06 ± 242.42 vs. 468.19 ± 91.25, P < 0.05) was significantly higher in the POTS-positive group compared to the HUTT-negative group (Table 2).

The instantaneous time-domain during HUTT results indicated that SDNN (91.69 ± 8.98 vs. 82.00 ± 7.46, P > 0.05), rMSSD (23 ± 2.88 vs. 24.46 ± 3.01, P > 0.05), SDSD (21.38 ± 3.38 vs. 24.46 ± 3.01, P > 0.05), pNN50 (4.38 ± 1.24 vs. 5.38 ± 1.41, P > 0.05) showed no statically significant differences between POST patients and HUTT-negative group. The instantaneous frequency-domain analysis during HUTT revealed no statistically significant differences between POTS patients and the HUTT-negative group across the evaluated parameters. Specifically, VLF (1.69 ± 0.15 vs. 1.47 ± 0.14, P > 0.05), LF (0.98 ± 0.11 vs. 1.15 ± 0.12, P > 0.05), HF (0.95 ± 0.09 vs. 1.12 ± 0.15, P > 0.05), and LF/HF (1.06 ± 0.08 vs. 1.18 ± 0.13) demonstrated no statistically significant differences (Table 3). The instantaneous time-domain and frequency-domain analysis eg SDNN, rMSSD, SDSD, pNN50, VLF, LF, HF demonstrated no statistically significant differences.

**Table 3:** Total heart rate variability analysis during head-up tilt test.

	Group1	Group 2	T-test/χ^2^ test	P value
SDNN (ms)	91.69 ± 8.98	82.00 ± 7.46	0.83	0.41
rMSSD (ms)	23 ± 2.88	24.46 ± 3.01	0.35	0.73
SDSD (ms)	21.38 ± 3.38	24.46 ± 3.01	0.68	0.50
pNN50 (%)	4.38 ± 1.24	5.38 ± 1.41	0.53	0.60
VLF	1.69 ± 0.15	1.47 ± 0.14	1.07	0.29
LF	0.98 ± 0.11	1.15 ± 0.12	1.04	0.31
HF	0.95 ± 0.09	1.12 ± 0.15	0.94	0.36
LF/HF	1.06 ± 0.08	1.18 ± 0.13	0.81	0.42

Group 1: patient with postural orthostatic tachycardia syndrome. Group 2: Patients experiencing syncope or presyncope exhibited negative results on the head-up tilt test. The standard deviation of all normal to normal NN intervals (SDNN), root mean square of successive differences between adjacent normal cycles (RMSSD), standard deviation of successive difference (SDSD), percentage of NN 50 in the total number of NN intervals (PNN50) are indicated. VLF: very low frequency, LF: low frequency, HF: high frequency, LF/HF: ratio of low frequency and high frequency.

## 4 Discussion

### 4.1 Overview of the study’s key findings

Postural Orthostatic Tachycardia Syndrome (POTS) was first characterized by Low and colleagues at the Mayo Clinic in 1993 (Schondorf and Low, 1993). POTS is diagnosed based on a sustained heart rate (HR) increase exceeding 30 beats per minute, or an HR reaching 120 beats per minute within 10 minutes of upright posture, in the absence of orthostatic hypotension (Stewart et al., 2018). Notably, while only approximately one-third of POTS patients experience episodes of syncope, most report daily occurrences of pre-syncope (Raj, 2013).

This study included 26 patients presenting with syncope or pre-syncope. Of these, 13 patients were diagnosed with POTS, while the remaining 13 patients without POTS had negative HUTT results. According to the pre-HUTT HRV by 24-hour Holter monitoring, Time-domain parameters of (HRV, including SDNN, SDANN, SDNN index, rMSSD, pNN50, and TINN, were significantly higher in patients with positive POTS diagnoses compared to those with negative HUTT results. Similarly, frequency-domain HRV parameters, such as low frequency (LF) and high frequency (HF), were significantly elevated in the POTS-positive group. However, no statistically significant differences were observed in the HRV parameters during HUTT between the two groups, the findings suggest that pre-HUTT HRV, as assessed by 24-hour Holter monitoring, may provide greater clinical relevance in distinguishing between these groups.

### 4.2 Clinical implication of heart rate variability

HRV is a noninvasive measure of the variability in the intervals between consecutive heartbeats and serves as an indicator of the balance between sympathetic and parasympathetic modulation of cardiac activity (Anon, 1996; Bai et al., 2009). HRV metrics are broadly categorized into time-domain and frequency-domain measures. Firstly, among time-domain parameters, the standard deviation of all normal-to-normal intervals (SDNN) is widely regarded as a measure of "global HRV," reflecting all cyclic components contributing to temporal variations in heartbeats. In this study, 24-hour Holter recordings revealed that SDNN values were significantly higher in patients with POTS compared to patients with negative results on the HUTT. These findings suggest that the total variance of HRV is greater in POTS patients than in the HUTT-negative group, highlighting differences in autonomic regulation between these populations. Secondly, the standard deviation of the averages of 5-minute NN intervals (SDANN) reflects long-term variations in heart rate and is influenced by changes in sympathetic tone, with an inverse relationship to sympathetic activity. An increase in SDANN corresponds to a decrease in sympathetic activity (Li and Zheng, 2022). In this study, 24-hour Holter recordings demonstrated significantly higher SDANN values in the POTS group compared to the HUTT-negative group, leading to the conclusion that patients with POTS exhibit reduced sympathetic activity. Thirdly, the root mean square of successive differences (rMSSD) is calculated as the square root of the mean of the squared differences between consecutive NN intervals, while the proportion of NN intervals differing by more than 50 ms (pNN50) is expressed as NN50 divided by the total number of NN intervals. Both rMSSD and pNN50 are measures of short-term HRV and reflect parasympathetic activity, with higher values indicating increased parasympathetic tone. In this study, rMSSD and pNN50 values were significantly higher in the POTS group compared to the HUTT-negative group, supporting the conclusion that parasympathetic activity is elevated in patients with POTS. Finally, frequency-domain parameters, particularly LF and HF power, are widely used in HRV analysis. Recent interpretations suggest that LF reflects the combined influence of both sympathetic and parasympathetic activation, while HF is primarily associated with parasympathetic activity. Higher HF power is indicative of increased parasympathetic nervous system activation. In this study, 24-hour Holter recordings using frequency-domain methods revealed that HF power was significantly higher in the POTS group compared to the HUTT-negative group. These findings further support the conclusion that patients with POTS exhibit heightened parasympathetic tone.

### 4.3 Comparison with existing literature

Luka et al. conducted an analysis of HRV in patients with hyperadrenergic versus non-hyperadrenergic POTS. Their study revealed that hyperadrenergic patients exhibited a higher supine heart rate, an elevated low-frequency/high-frequency (LF/HF) ratio, and reduced high-frequency (HF) values during supine phase. However, no significant differences were observed in any HRV parameters during the tilted phase between the hyperadrenergic and non-hyperadrenergic POTS groups (Crnošija et al., 2016). In our study, we did not identify a significant difference in the heart rate variability (HRV) parameters between the POST group and the control group during HUTT. This discrepancy may be attributed to the different HRV measurement periods analyzed in our research. In our analysis, we assessed heart rate variability (HRV) during the tilt test in its entirety, without separately evaluating the HRV parameters for the supine and tilted phases. In future studies, we aim to perform a comprehensive analysis of heart rate variability, examining both the overall response and specific phases, such as the supine and tilted phases, as well as other relevant heart rate intervals, in patients undergoing the HUTT.

Wang et al. conducted a comparative analysis of baseline HRV parameters collected before the HUTT in a cohort of patients with POTS and a healthy control group. Their findings revealed that the SDNN index, rMSSD, pNN50, TR index, and LF components were significantly elevated in the POTS group compared to controls (Wang et al., 2019). These results align closely with those observed in our study. According to previous literature, neuropathic POTS is primarily characterized by enhanced vasodilation and vagal tone. Wang et al. also noted an increase in vagal nerve tone in the POTS group compared to the control group. This change in vagal nerve tone aligns with the results observed in our study. However, they did observe a significant decreased in the SDANN values between the POTS and control groups. This variation may be attributed to the heterogeneity of the patient populations across different studies. In the subsequent phase of the study, we aim to enlarge the sample size to facilitate more robust and detailed correlation analyses.

Sumiyoshi highlighted that power spectral analysis of HRV revealed an increase in the low-frequency to high-frequency (LF/HF) ratio, corresponding to the onset of orthostatic intolerance (Sumiyoshi et al., 1999). In our study, we observed no statistically significant difference in the LF/HF ratio between the POTS group and the HUTT-negative group. This discrepancy may be attributable to differences in the timing of HRV parameter assessment. Specifically, we analyzed HRV parameters prior to the HUTT, whereas Sumiyoshi evaluated the LF/HF ratio during the HUTT itself. The author only analyzed the changes in frequency analysis of holter recording during HUTT in one POTS patient, and no negative control group was compared.

Orjatsalo et al. highlighted that the high-frequency attenuated in both POTS group and control group, but the attenuation was bigger in POTS group (Orjatsalo et al., 2020). In our study, we observed an elevated HF component prior to the HUTT in POTS group and unchangeable HF component during HUTT in POTS group; however, discrepancies in the parameter may be attributed to heterogeneity of patient included and variations in the timing period of HUT observations.

### 4.4 Physiological mechanisms of POTS and interpretation

The maintenance of adequate blood flow to vital organs during the transition to an upright posture presents a significant physiological challenge. This process requires a delicate balance between neural, hormonal, and hemodynamic adjustments to sustain sufficient atrial pressure. The sympathetic nervous system (SNS) plays a crucial role as a rapid-acting regulatory mechanism, enabling the cardiovascular system to adapt to postural changes. Its activity is predominantly modulated by mechanoreceptors, which detect changes in pressure, and to a lesser extent, by chemoreceptors. High-pressure mechanoreceptors, known as arterial baroreceptors, are located in the carotid sinus and aortic arch, whereas low-pressure mechanoreceptors, or cardiopulmonary baroreceptors, are found in the great veins, atria (Bainbridge reflex), and ventricles (Bezold-Jarisch reflex). These stretch-activated ion channels influence sympathetic outflow. In humans, the normal baroreflex response to reduced venous return during upright posture involves an increase in vascular sympathetic nerve activity and a decrease in parasympathetic activity (Chapleau et al., 1995, Vasquez et al., 1997). This response leads to elevated heart rate and contractility as well as increased peripheral vasoconstriction, thereby maintaining atrial pressure and preserving cerebral perfusion. These mechanisms are essential for preventing symptoms such as those observed in patients with recurrent syncope or POTS. Schondorf and Low conducted a review of patients aged 20–51 who exhibited POTS during HUTT. They noted that increased catecholamine levels were observed in some of these patients (Schondorf and Low, 1993). Research from the Mayo Clinic regarding POTS revealed that at least 50 % of patients exhibit neuropathic features, approximately one-third present with hyperadrenergic characteristics, and one in seven demonstrates serological evidence suggestive of an autoimmune etiology (Low et al., 2009). Additionally, Lambert identified a reduction in the expression of norepinephrine transport proteins in patients with POTS (Lambert et al., 2008). POTS is classified as a variant of autonomic cardiovascular disorder; however, its precise etiology remains largely unknown. Despite its unclear origins, POTS is frequently debilitating, highlighting the need for further research into its underlying mechanisms and potential treatments.

Based on the relationship between HRV and autonomic nervous system (ANS) function, we concluded that patients with POTS exhibit decreased sympathetic nervous system activity alongside increased parasympathetic nervous system activity. This imbalance likely results in impaired sympathetically mediated vasoconstriction, leading to reduced cerebral blood flow. The sympathetic nervous system plays a critical role in the regulation of lower limb veins through α-adrenergic receptors (Blöchl-Daum et al., 1991). Activation of these receptors by norepinephrine release induces smooth muscle contraction in the venous walls, which facilitates venoconstriction. This physiological mechanism enhances venous return, optimizing blood flow back to the heart and contributing to increased cardiac output, particularly during hemodynamic challenges such as postural changes. In our study, we found POTS exhibit reduced sympathetic activity. This impaired sympathetic neuropathy may lead to inability of the peripheral vascular to maintain adequate vascular resistance in orthostatic position. This results in an abnormally pronounced pooling of blood in dependent regions of the body, including the lower extremities, forearms, and mesenteric vasculature, during upright posture. The redistribution of blood away from the central vasculature triggers a compensatory response characterized by an increase in heart rate and myocardial contractility, aimed at preserving adequate cerebral perfusion. Such alterations in autonomic function are insufficient to adequately counteract hemodynamic changes, contributing to the clinical manifestations of POTS. Consequently, diminished sympathetic activity and elevated parasympathetic activity are identified as key contributors to the pathophysiology of POTS, which may explain the frequent occurrence of syncope and presyncope in these patients.

## 5 Limitation

This retrospective study was conducted at a single institution, and its findings should be interpreted with caution due to the limited sample size. Nevertheless, this work represents a rare original contribution to the evaluation of HRV both Pre-HUTT and instantaneous-HUTT in patients with POTS compared to healthy controls. We performed comprehensive analyses utilizing both time-domain and frequency-domain methodologies. Simultaneously, we investigated the underlying cause of POTS symptoms with a focus on the potential role of sympathetic nerve dysfunction in patients with POTS. To confirm and extend these findings, future research should adopt a prospective design and include a substantially larger cohort of participants.

## 6 Conclusion

The findings related to HUTT and HRV in POTS patients show considerable heterogeneity. Our study showed diminished sympathetic activity and elevated parasympathetic activity are identified as key contributors to the pathophysiology of POTS, which may explain the frequent occurrence of syncope and presyncope in these patients. Our findings also indicate that pre-HUTT HRV, assessed via 24-hour Holter monitoring, may be more clinically relevant for distinguishing between these groups. In the future, we should recruit more POTS patients and further study the related heart rate variability and evaluate the mechanism.

## CRediT authorship contribution statement

**Li JingXiu:** Writing – original draft, Data curation. **Gao Min:** Writing – review & editing. **Qiu Xin:** Writing – original draft, Formal analysis.

## Ethics

Ethical approval for this study was granted by the ethics committee of the First Affiliated Hospital of USTC, in accordance with established ethical guidelines, including the Declaration of Helsinki. Written informed consent was obtained from all participants [2024-RE-468].

## Author contributions

Jing-Xiu Li and Xin Qiu contributed significantly to manuscript preparation. Jing-Xiu Li and Min Gao performed the analysis with result and discussion. All authors concur on the sequence of their names in the manuscript.

## Funding

This study was supported by Anhui Provincial Health Research Project (No. AHWJ2022a008). The funders had no role in the study design, data collection and analysis, decision to publish, or preparation of the manuscript.

## Conflicts of Interest

The authors declare no conflicts of interest.

## Data Availability

Data may be acquired by communicating to the corresponding author.
